# Genetic characterization of Chikungunya virus from New Delhi reveal emergence of a new molecular signature in Indian isolates

**DOI:** 10.1186/1743-422X-9-100

**Published:** 2012-05-25

**Authors:** Jatin Shrinet, Shanu Jain, Anil Sharma, Shashi Shekhar Singh, Kalika Mathur, Vandita Rana, Raj K Bhatnagar, Bhupendra Gupta, Rajni Gaind, Monorama Deb, Sujatha Sunil

**Affiliations:** 1 Insect Resistance Group, International Centre for Genetic Engineering and Biotechnology, New Delhi, India; 2 Vardhman Mahavir Medical College & Safdarjung Hospital, New Delhi, India

**Keywords:** Chikungunya virus, India, Molecular signatures, ECSA lineage, Asian lineage

## Abstract

**Background:**

Chikungunya (CHIK) is currently endemic in South and Central India and exist as co-infections with dengue in Northern India. In 2010, New Delhi witnessed an outbreak of CHIK in the months October-December. This was the first incidence of a dominant CHIK outbreak in Delhi and prompted us to characterize the Delhi virus strains. We have also investigated the evolution of CHIK spread in India.

**Findings:**

Clinical samples were subjected to RT-PCR to detect CHIK viral RNA. The PCR amplified products were sequenced and the resulting sequences were genetically analyzed. Phylogenetic analysis based on partial sequences of the structural proteins E1 and E2 revealed that the viruses in the latest outbreak exhibited ECSA lineage. Two novel mutations, E1 K211E and E2 V264A were observed in all Delhi isolates. In addition, CHIKV sequences from eight states in India were analyzed along with Delhi sequences to map the genetic diversity of CHIKV within the country. Estimates of average evolutionary divergence within states showed varying divergence among the sequences both within the states and between the states. We identified distinct molecular signatures of the different genotypes of CHIKV revealing emergence of a new signature in the New Delhi clade. Statistical analyses and construction of evolutionary path of the virus within the country revealed gradual spread of one specific strain all over the country.

**Conclusion:**

This study has identified unique mutations in the E1 and E2 genes and has revealed the presence of ancestral CHIKV population with maximum diversity circulating in Maharashtra. The study has further revealed the trend of CHIK spread in India since its first report in 1963 and its subsequent reappearance in 2005.

## Findings

Chikungunya (CHIK), a neglected vector borne disease until as few years ago, has grown to pandemic levels, with hundreds of thousands cases reported from all around the globe. In India, the infection reemerged in seven states in 2005 and by the latest report in 2010, has spread to more than 18 states/Union Territories within the country affecting more than 3.7 million individuals [1]. Moreover, the number of cases has been grossly under reported due to mistaken diagnosis of dengue and non-reporting of suspected cases and related deaths thereby making the number of CHIK infection much more than reported [2]. Since its reemergence, the intensity of the infection has increased with every passing year with 45%–63% attack rates in several areas during outbreaks [3,4]. India is endemic to dengue fever and due to overlapping symptoms; CHIK infection is generally mistaken to be the former thereby leading to misdiagnosis. Persistent chronic phase is sometimes used as a distinguishing feature between dengue and CHIK [5].

Delhi witnessed a CHIK outbreak between October-December 2010 just after experiencing an outbreak of dengue. Even though CHIK has been occurring in Delhi since 2006 as co-infections with dengue [6] and has not been known to cause major outbreaks, last few months of year 2010 saw an unprecedented increase in number of affected CHIK cases, prompting us to characterize the strains and study the infection with special attention to its divergence in the country since the first outbreak in 1963.

Blood samples were drawn from 289 patients, who were clinically suspected to have CHIK, over a period of three months since the onset of the outbreak. The blood samples were received from outpatient and inpatients departments as part of the routine laboratory investigations for suspected cases that were suffering from the classical symptoms of fever, polyarthralgia, myalgia and skin rashes. These patients belonged to different localities of Delhi. The sera was separated and stored at −80°C. All sera samples were subjected to ELISA for testing the presence of Chikungunya virus (CHIKV) IgM antibodies using the IgM capture ELISA kits supplied by National Institute of Virology (NIV), Pune to Government Hospitals during the outbreak. A total of 80 sera samples were used for the study. The sera were not subjected to passaging using any cell lines to ensure the sequence integrity of the strains.

Viral RNA was extracted directly from sera using the High Pure Viral Nucleic Acid kit (Roche, Germany) according to manufacturer’s instructions. CHIKV structural genes E1 and E2 were analyzed for the present study. Complete gene of E1 (1320 bps) and E2 (1266 bps) and partial region (555 bps) of the E1 structural gene were amplified using new primer sets as well as previously published primers [7,8] with modifications. For amplifying partial region of E1 gene, a nested RT-PCR protocol was developed to increase the sensitivity of RT-PCR in the clinical samples. All PCR primer sets used in the study and their genome positions are listed in Table 1. The amplicons were purified using Qiaquick PCR purification kit (Qiagen, USA) and sequenced using both forward and reverse primers. All sequences were trimmed, aligned and analyzed.

**Table 1:** Primer sets used in study

**Primer Name**	**Sequence 5′-3′**	**Genome position**	**Amplicon size bps**	**Reference**	**Where used in study**
E1-primary-F	ACAAAACCGTCATCCCGTCTC	10145–11158	1013	[7]	Primary PCR
E1-primary-R	TGACTATGTGGTCCTTCGGAGG				
E1-F1	GCTCCGCGTCCTTTACC	10389–10943	555	[8]	Nested PCR, Phylogenetic analysis
E1-R1	ATGGCGACGCCCCCAAAGTC				
E1-gene-F	GCGTACGAACACGTAACA	9991–11310	1320	This study	Molecular signatures
E1-gene-R	GTGCCTGCTGAACGACAC				
E2-gene-F	AGCACCAAGGACAACTTCAAT	8542–9807	1266	This study	Phylogenetic analysis Molecular signatures
E2-gene-R	TTTAGCTGTTCTGATGCAGC				

Phylogenetic analyses, molecular evolution analyses of E1 and E2 genes were performed using MEGA 5 [9] and DNAsp softwares [10]. As references, strains of the three genotypes, namely ESCA, Asian and African [11] were taken based on their sampling dates and low passage history ( Additional file 1: Table S1). Pair-wise genetic divergences within group and net evolutionary divergence between groups among the sequences were calculated following the maximum composite likelihood model. The sequences were further analyzed to obtain a Neighbor Joining tree with bootstrap re-sampling (1000 replicates) and validated using other distance based and character based tree models. Neutrality of mutation in the sequences was determined using Tajima’s D test [12] and validated using Fu & Li D* & F* [13]. Network analysis was performed based on SplitsTree 4 (Ver 4.11.3) [14] to generate a hypothetical evolutionary pathway of 108 Indian CHIKV samples in India since the 1963 epidemic till the latest outbreak in 2010.

Of the total 80 samples used in the study, 56 samples were ELISA positive and 22 samples were ELISA negative. ELISA status of remaining two samples was not known. A total of 36 samples (44%) showed amplification of the partial gene of E1. No-RT control RNA and RNA from healthy volunteer served as negative control. Of these PCR positive samples, 53% (n = 19) of samples were negative by ELISA, 42% (n = 15) of samples were positive by both methods and ELISA status of two samples (5%) was not known. Three samples were negative both for ELISA and RT-PCR and were excluded from further analysis. Using the nested RT-PCR protocol, viral RNA was detected upto Day 11 from the onset of clinical symptoms (data not shown) making this an attractive alternative to the existing RT-PCR detecting methods. Partial region of E1 gene (555 bps) from 36 samples were sequenced and aligned to study sequence variation. For the purpose of generating the molecular signature for the E1 gene, the complete gene was amplified and sequenced from seven clinical isolates. A subset of positive samples (n = 14) were used to amplify E2 gene. The amplified products were purified, sequenced and phylogenetic analysis performed.

Nucleotide sequence analysis was performed on partial regions of E1 and E2 genes on all Delhi strain sequences and compared with the 1953 Tanzanian strain (Accession no: 045811.1). With respect to E1 gene, at the nucleotide level, all Delhi samples showed variations at 15 nucleotide positions producing both synonymous and non-synonymous mutations (Table 2). Of the synonymous mutations, one at position C10602T was found to be unique to all Delhi strains. Furthermore, six strains showed additional variations at the nucleotide level. At the amino acid level, three amino acid substitutions, namely, E1 K211E, E1 M269V and E1 D284E were seen in all samples (Table 2). Apart from the above mentioned variations, three samples showed unique amino acid changes, namely, IND-10-DEL48 at amino acid positions E1 V179A, E1 S234P, IND-10-DEL88 at position E1 R196K, IND-10-DEL108 at position E1 R247C (Table 2). Analysis of E2 gene at the nucleotide level revealed thirty two variations in all Delhi samples when compared with 1953 Tanzanian strain (Table 3). Of these nucleotide variations, eight resulted in amino acid changes. Furthermore, samples IND-10-DEL10 and IND-10-DEL15 revealed unique amino acid variations at position E2 V50A and E2 C389R respectively (Table 3).

**Table 2:** Nucleotide and amino acid variations of E1 gene in Delhi samples in comparison with ECSA lineage and other Indian samples are shown here

**Nucleotide Position**	**Amino acid variation**	**Samples**									
		**ECSA#**	**Indian Samples**	**All Delhi Sample**	**IND-10-DEL4**	**IND-10-DEL17**	**IND-10-DEL48**	**IND-10-DEL88**	**IND-10-DEL91**	**IND-10-DEL100**	**IND-10-DEL108**
**10521**		**A**	**.**	**.**	**.**	**.**	**.**	**G**	**.**	**.**	**.**
**10524**		**C**	**T/C**	**T**							
10529*	**V179A**	**T**	**.**	**.**	**.**	**.**	**C**	**.**	**.**	**.**	**.**
**10545**		**T**	**C**	**C**							
**10548**		**C**	**T/C**	**T**							
10580*	**R196K**	**G**	**.**	**.**	**.**	**.**	**.**	**A**	**.**	**.**	**.**
**10602**		**C**	**.**	**T**	**C**						
**10614**		**G**	**A/G**	**A**							
**10623**		**C**	**T/C**	**T**							
10624*	**K211E**	**A**	**A/G**	**G**							
**10641**		**C**	**T/C**	**T**							
**10665**		**G**	**.**	**.**	**.**	**.**	**A**	**.**	**.**	**.**	**.**
**10689**		**A**	**A/G**	**.**	**.**	**G**	**.**	**.**	**T**	**.**	**.**
10693*	**S234P**	**T**	**.**	**.**	**.**	**.**	**C**	**.**	**.**	**.**	**.**
**10723**		**T**	**C**	**C**							
10732*	**R247C**	**C**	**.**	**.**	**.**	**.**	**.**	**.**	**.**	**.**	**T**
**10734**		**A**	**C/A**	**C**							
**10743**		**G**	**A/G**	**A**							
**10746**		**A**	**G**	**G**							
10798*	**M269V**	**A**	**G/A**	**G**							
10845*	**D284E**	**T**	**A/T/C**	**A**							
**10848**		**G**	**.**	**.**	**.**	**.**	**.**	**.**	**.**	**A**	**.**
**10893**		**A**	**G**	**G**							

Asterisk next to nucleotide position denotes non-synonymous mutations. Dot represents same nucleotide as ECSA. Blank represents same nucleotide as All Delhi samples.

#ECSA strains (Accession nos. AF369024.2 and HM045811.1).

**Table 3:** Nucleotide and amino acid variations of E2 gene in Delhi samples in comparison with ECSA lineage and other Indian samples are shown here

**Nucleotide Position**	**Amino acid variation**	**Samples**				
		**ECSA#**	**Indian Samples**	**All Delhi Samples**	**IND-10-DEL10**	**IND-10-DEL15**
8690*	**V50A**	**T**	**.**	**.**	**C**	**.**
**8703**		**T**	**C**	**C**		
**8721**		**T**	**C/T**	**C**		
**8727**		**T**	**C/T**	**C**		
**8748**		**C**	**T/C**	**T**		
**8757**		**T**	**C/T**	**C**		
**8784**		**C**	**G/T**	**G**		
**8811**		**A**	**G**	**G**		
**8814**		**C**	**T/C**	**T**		
**8859**		**G**	**A/G**	**A**		
**8865**		**A**	**G/A**	**G**		
**8910**		**C**	**T/C**	**T**		
**8952**		**C**	**T/C**	**T**		
9031*	**A164T**	**G**	**A/G**	**A**		
**9081**		**G**	**A/G**	**A**		
**9138**		**T**	**C**	**C**		
9173*	**I211T**	**T**	**C**	**C**		
**9270**		**G**	**.**	**.**	**A**	**.**
**9276**		**C**	**T**	**T**		
**9285**		**C**	**T/C**	**T**		
9332*	**V264A**	**T**	**T**	**C**		
9437*	**S299N**	**G**	**A**	**A**		
9476*	**T312M**	**C**	**T/C**	**T**		
**9480**		**C**	**T**	**T**		
**9489**		**G**	**A/G**	**A**		
**9525**		**T**	**C**	**C**		
9571*	**A344T**	**G**	**A**	**A**		
**9588**		**C**	**T/C**	**T**		
**9609**		**C**	**T**	**T**		
**9610**		**T**	**C/T**	**C**		
**9615**		**C**	**T**	**T**		
9664*	**S375T**	**T**	**A/T**	**A**		
9698*	**V386A**	**T**	**C/T**	**C**	**.**	**C**
**9706**	**C389R**	**T**	**.**	**.**		
**9741**		**C**	**T/C**	**T**		

Asterisk next to nucleotide position denotes non-synonymous mutations. Dot represents same nucleotide as ECSA. Blank represents same nucleotide as All Delhi samples.

Of the variations in both E1 and E2 genes, two amino acid residues E1 K211E and E2 V264A deserves special mention. The E1 K211 mutation has been reported earlier in the Asian lineage and the ECSA lineage shows E1 K211N [15]. The E1 K211E variation in ECSA lineage has been reported in one sample from Puducherry [16]. In the present study, all the samples collected in Delhi have shown this variation. Tsetsarkin and co-workers [17] recently studied the involvement of E1-211 in the adaptation of the Asian strain and eventually in its spread. They illustrated that E1-211 along with another mutation at position E1-98 were associated with CHIKV sensitivity to *Ae. albopictus*-adaptive effect of E1 A226V mutation. Similarly, in E2, amino acid variation E2 V264A was seen in all samples of Delhi population. Both the E1 K211E and E2 V264A variations have also been reported in the autochthonous imported case in France from India during the same period in 2010 from a different region [18] as well as from cases in Tamil Nadu and Andhra Pradesh [19].

Furthermore, critical evaluation of the complete E1 and E2 gene sequences data allowed us to generate a molecular signature for the specific genotypes of CHIKV. The molecular signature illustrate that the Delhi samples showed a different signature from those seen in the reference strains and other strains from India reported since 1963 and the latest documented epidemic in 2005 onwards (Tables 4 &5). A recent study has reported emergence of a novel subgroup among CHIKV strains in Asian countries; however, homologous recombination did not contribute to the genetic diversity of these strains [20]. It should be noted that the above study did not include any of the strains from the latest outbreak in 2010 in India. Molecular signatures identified by our study raises a possibility of recent recombination events in the Indian CHIKV population and warrant further detailed analysis.

**Table 4:** Molecular signatures derived on amino acid residue variations in chikungunya whole E1 proteins are shown below

**Collection Year**	**Sample**		**E1 protein: Amino Acid Residue Position**												
		**72**	**98**	**142**	**145**	**162**	**211**	**225**	**226**	**269**	**276**	**284**	**296**	**315**	**322**
**1966/2005**	**Africa**	**S**	**A**	**I**	**A**	**V**	**K**	**A**	**A**	**I/V**	**I**	**D**	**V**	**V**	**A**
**1953**	**ECSA**	**N**	**A**	**I**	**T**	**I**	**K**	**A**	**A**	**M**	**M**	**D**	**L**	**V**	**A/V**
**1985/2007**	**Asia**	**S**	**T**	**I/V**	**A/S**	**I**	**E**	**S**	**A**	**M**	**M**	**D**	**L**	**V**	**A**
**2010**	**Delhi**♦	**N**	**A**	**I**	**T**	**I**	**E**	**A**	**A**	**V**	**M**	**E**	**L**	**V**	**A**
**1963**	**West Bengal**	**S**	**T**	**V**	**S**	**I**	**E**	**S**	**A**	**M**	**M**	**D**	**L**	**A**	**A**
**2003/2006**	**Maharashtra**	**N/S**	**A/T**	**I/V**	**T/S**	**I**	**K/E**	**A/S**	**A**	**V/M**	**M**	**E/D**	**L**	**V/A**	**A**
**2006**	**Gujarat**	**N**	**A**	**I**	**T**	**I**	**K/N**	**A**	**A**	**V**	**M**	**E**	**L**	**V**	**A**
**2006**	**Puducherry**	**N**	**A**	**I**	**T**	**I**	**K/E**	**A**	**A**	**V**	**M**	**E**	**L**	**V**	**A**
**2006/2007/2008**	**Kerala**	**N**	**A**	**I**	**T**	**I**	**K**	**A**	**A/V**	**V**	**M**	**E**	**L**	**V**	**A**
**2007**	**Uttar Pradesh**	**N**	**A**	**I**	**T**	**I**	**K**	**A**	**A**	**V**	**M**	**E**	**L**	**V**	**A**
**2006/2008**	**Karnataka**	**-**	**A**	**I**	**T**	**I**	**K**	**A**	**A/V**	**V**	**M**	**E**	**L**	**V**	**A**
**2008/2009**	**Andhra Pradesh**	**N**	**A**	**I**	**T**	**I**	**K/N**	**A**	**A**	**V**	**M**	**E**	**L**	**V**	**A**

Amino acid residue positions of the complete E1 proteins, the Country/State and year of collection of CHIKV are denoted. (−) denotes sequence information not available.

**Table 5:** Molecular signatures derived on amino acid residue variations in chikungunya whole E2 proteins are shown below

**Collection Year**	**Sample**	**E2 protein: Amino Acid Residue Position**														
		**2**	**118**	**149**	**157**	**205**	**211**	**255**	**264**	**299**	**317**	**318**	**344**	**375**	**384**	**386**
**1966/2005**	**Africa**	**T**	**S**	**K**	**V**	**G**	**T**	**I**	**V**	**N**	**V**	**T**	**T**	**S**	**T**	**V**
**1953**	**ECSA**	**T**	**S**	**K**	**V**	**G**	**I**	**I**	**V**	**S**	**V**	**V**	**A**	**S**	**M**	**V**
**1985/2007**	**Asia**	**I**	**G**	**R**	**A**	**D**	**T**	**V**	**V**	**N**	**I**	**R**	**T**	**S**	**V**	**V**
**2010**	**Delhi**♦	**T**	**S**	**K**	**V**	**G**	**T**	**I**	**A**	**N**	**V**	**V**	**T**	**T**	**M**	**A**
**1963**	**West Bengal**	**I**	**G**	**R**	**A**	**D**	**T**	**I**	**V**	**N**	**V**	**R**	**T**	**S**	**V**	**V**
**2003/2006**	**Maharashtra**	**I/T**	**G/S**	**K**	**V**	**D/G**	**T**	**I**	**V**	**N**	**V**	**R/V**	**T**	**S/T**	**V/M**	**V/A**
**2006**	**Gujarat**	**T**	**S**	**K**	**V**	**G**	**T**	**I**	**V**	**N**	**V**	**V**	**T**	**T**	**M**	**A**
**2006/2007/2009**	**Kerala**	**T**	**S**	**K**	**V**	**G**	**T**	**I**	**V**	**N**	**V**	**V**	**T**	**T**	**M**	**A**
**2006**	**Rajasthan**	**T**	**S**	**K**	**V**	**G**	**T**	**I**	**V**	**N**	**V**	**V**	**T**	**T**	**M**	**A**
**2006**	**Tamil Nadu**	**T**	**S**	**K**	**V**	**G**	**T**	**I**	**V**	**N**	**V**	**V**	**T**	**T**	**M**	**A**
**2006**	**Karnataka**	**T**	**S**	**K**	**V**	**G**	**T**	**I**	**V**	**N**	**V**	**V**	**T**	**T**	**M**	**A**
**2006/2008/2009**	**Andhra Pradesh**	**T**	**S**	**K**	**V**	**G**	**T**	**I**	**V**	**N**	**V**	**V**	**T**	**T**	**M**	**A**

Amino acid residue positions of the complete E2 proteins, the Country/State and year of collection of CHIKV are denoted. (−) denotes sequence information not available.

**Figure 1:**
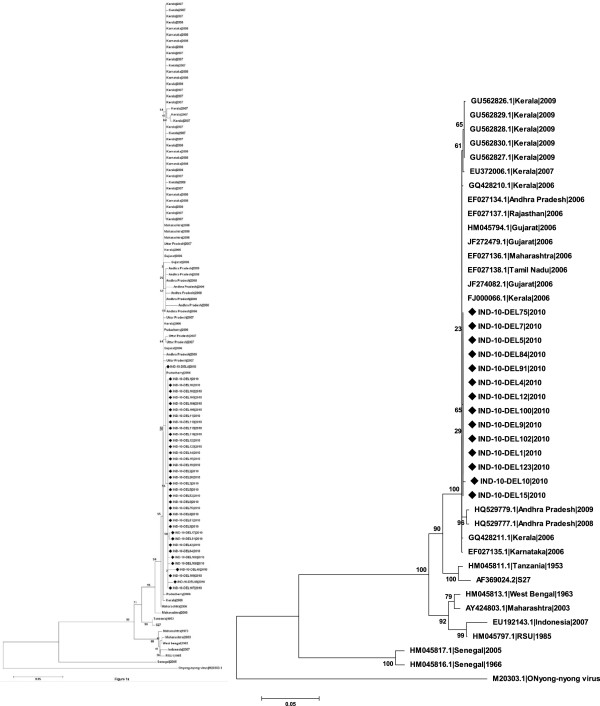
**A & B: Phylogenetic relationship among CHIKV isolates from eight states of India. A)** based on partial E1 gene sequences (428 nt) performed on 109 nucleotide sequences and **B)** on partial E2 gene sequences (1002 nt) performed on 42 nucleotide sequences. The tree was constructed using Neighbor-Joining method. The percentage bootstrap support values (1000 replicates) are shown next to the branches. The tree is drawn to scale, with branch lengths in the same units as those of the evolutionary distances used to infer the phylogenetic tree. The evolutionary distances were computed using the Kimura 2-parameter method and are in the units of the number of base substitutions per site. Evolutionary analyses were conducted in MEGA5. All sequences of strains sequenced for this study has been submitted in GenBank; other sequences were retrieved from GenBank. Scale bar indicates number of base substitutions per site. Onyong-nyong virus used as out-group. Isolates sequenced in this study are indicated by ‘♦’.

Phylogenetic analysis based on E1 of all Delhi samples with strains previously reported from eight states in India since 1963 (n = 109) revealed that all Delhi samples were of ECSA lineage and formed a single subclade within the Indian sequences (Figure 1a). Similar result was seen with E2 gene (n = 42) (Figure 1b). Evolutionary Divergence over 102 sequence pairs for E1 gene was calculated with a total of 143 positions in the final dataset and sequences from Maharashtra were found to be most divergent among all sequences analyzed. Tests of neutrality determined by Tajima’s D test showed positive selection in E1 gene, that was further validated by Fu & Li’s D* and F* tests (*p* < 0.05). Previous studies [15] have shown that E1 K211 is a positively selected site with a posterior probability of >75%. Another very recent study conducted on samples collected from different regions in south India, also showed that E1 K211E mutation is being positively selected [19]. With respect to fitness of these strains in the vector, it should be noted that Delhi had traditionally predominance of *Ae. aegypti* population; however, it has been shown recently that *Ae. albopictus* is adapted to urban breeding conditions and also plays a role in dengue transmission in Delhi and National Capital Territory of Delhi (NCT) [21]. It is noteworthy that *Ae. aegypti* is very prevalent in Tamil Nadau and Andhra Pradesh from where the samples in Sumathy et al., study [19] were taken while *Ae. albopictus* density is high in the neighbouring states. Since it has been shown in previous studies [22] that mutations in E2 play an epistatic role on E1 gene, it will be very interesting to analyze the effect of these mutations with respect to vector adaptability and infectivity in the presence and absence of E1 A226V mutation which also is positively selected [19]. Estimates of average evolutionary divergence (AED) over sequence pairs within the states showed varying divergence among the sequences both within the states and between the states. Sequences derived from samples from Karnataka and Uttar Pradesh showed least divergence, while Maharashtra showed maximum divergence suggesting existence of ancestral population in Maharashtra.

**Figure 2:**
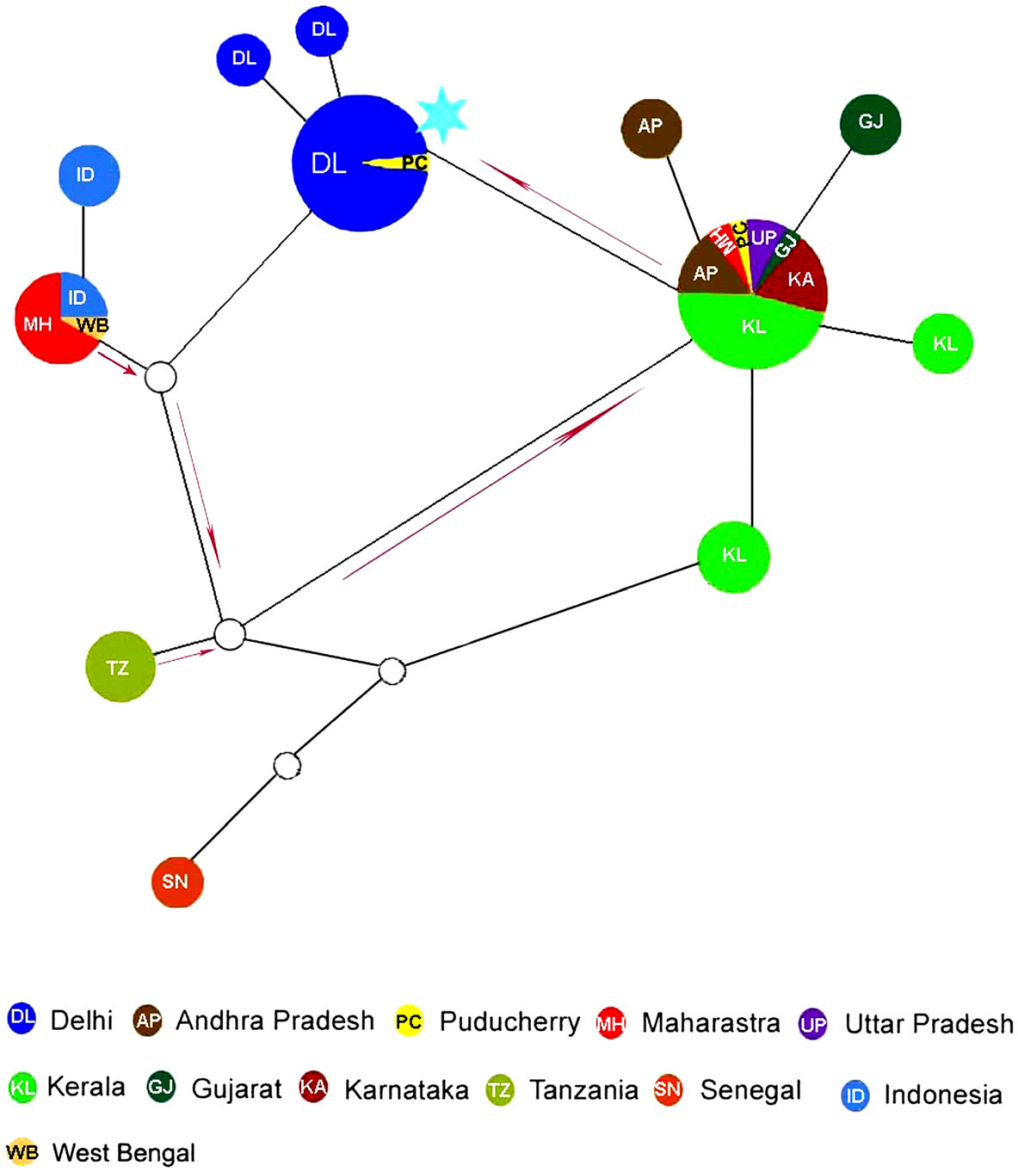
**Pruned quasi-median network of CHIKV partial E1 gene sequences.** 428 nucleotides of E1 gene (n = 108) was used to generate the network, here a specific color code are assigned to each state/Nation and diameter of nodes corresponds to the sample size within each node. The edges joining the nodes are not in scale. Hypothetical ancestral strains or strains not sampled are also represented as small nodes white in color. The proposed evolutionary path is shown by red arrows. Sky blue star represents substitution E1 K211E.

Following phylogenetic analysis and genetic diversity analyses, attempts were taken to construct a network on the basis of the important mutational events of the virus along its geographical spread along with the sampling date. The purpose of the network was to visualize the mutational paths based on the variations in the sequence analyzed as has been ascertained in recent studies [23,24]. In the present study, the mutation status combined with sampling date and geographical information provides insight in the spread of the strains with the country since the first incidence of the infection. Since the software also predicts hypothetical ancestral variants, this tool is useful to predict possible mutational steps in the absence of sampling. The network created using E1 gene sequences (n = 108) from different states of India shows evidence of possibly an independent introduction of CHIKV strains of Asian and ECSA lineage into the country with the Asian lineage introduced first followed by the ECSA lineage (Figure 2) also been discussed in previous reports [25]. The network also clearly shows Delhi strains forming a separate sub-clade along with one strain from Puducherry thereby indicating population amplification of the virus from Puducherry in Delhi. All our analyses were performed on partial E1 gene due to the availability of maximum number of sequences in this gene region (n = 108). To validate our result, we generated the network on complete gene of E1 gene with six strains from Delhi and 38 strains from other parts of India. The results corroborated with the network derived from the partial E1 gene sequences (data not shown).

In summary, our study based on the recent outbreak in Delhi has revealed important insights to the nature of the spread of CHIKV within the country. Even though CHIKV of the latest outbreak displayed ECSA lineage, there is emergence of a distinct molecular signature within the strains emphasizing that accumulation of mutations in the viral genome is leading to appearance of new subgroups and suggests a dynamic evolution of the virus [18]. Understanding the consequences of such changes in viral genomes is vital to prepare for any public health disaster like a serotype change or more serious clinical implications. Our attempts to trace the path of Indian CHIKV has also revealed that ECSA lineage may have emerged in India much later than the Asian lineage and have flared up along with the Reunion Island pandemic. Our study further shows evidence that important mutational events are also taking place within this clade that may have a potential role in the evolution of the virus and necessitates further detailed studies.

## Abbreviations

CHIKV, Chikungunya virus; ECSA, Eastern, central and South Africa; ELISA, Enzyme-linked immunosorbent assay; RT-PCR, Reverse transcriptase polymerase chain reaction.

## Competing interests

The authors declare that they have no competing interests.

## Authors’ contribution

SJ, VR, SSS, KM carried out viral RNA isolation, RT PCR and sequencing studies. SS, JS and AS carried out all genetic analysis. Authors from Vardhman Mahavir Medical College & Safdarjung Hospital were involved in CHIKV diagnosis, serology and sample collection. SS and RKB conceived the study and drafted the manuscript. SS and JS were involved in compiling the report. All authors read and approved the final version.

## Supplementary Material

Additional file 1 ** Table S1.** CHIKV sequences used in the study with accession number and collection date. Samples used in the study have been indicated by ‘♦’. Click here for file

## References

[B1] Ministry_of_Health_and_Family_Welfare Annual Report 2009–10 2010 Government of India: Ministry of Health and Family Welfare

[B2] Byahatti SM Dandagi GL Chikungunya-An update Journal of Clinical and Diagnostic Research 2010 4 3594 3598

[B3] Mavalankar D Shastri P Raman P Chikungunya epidemic in India: a major public-health disaster Lancet Infect Dis 2007 7 306 307 10.1016/S1473-3099(07)70091-9 17448932

[B4] Dwibedi B Mohapatra N Beuria MK Kerketta AS Sabat J Kar SK Rao EV Hazra RK Parida SK Marai N Emergence of Chikungunya virus infection in Orissa, India Vector Borne Zoonotic Dis 2009 10 4 347 354 19874187 10.1089/vbz.2008.0190

[B5] Robinson MC An epidemic of virus disease in Southern Province Tanganyika Territory, in 1952–53. I. Clinical features Trans R Soc Trop Med Hyg 1955 49 28 32 10.1016/0035-9203(55)90080-8 14373834

[B6] Chahar HS Bharaj P Dar L Guleria R Kabra SK Broorl Co-infections with chikungunya virus and Dengue virus in Delhi, India Emerg Infect Dis 2009 15 1077 1079 10.3201/eid1507.080638 19624923 PMC2744227

[B7] Santhosh SR Dash PK Parida MM Khan M Tiwari M Lakshmana Rao PV Comparative full genome analysis revealed E1: A226V shift in 2007 Indian Chikungunya virus isolates Virus Res 2008 135 36 41 10.1016/j.virusres.2008.02.004 18384900

[B8] Niyas KP Abraham R Unnikrishnan RN Mathew T Nair S Manakkadan A Issac A Sreekumar E Molecular characterization of Chikungunya virus isolates from clinical samples and adult Aedes albopictus mosquitoes emerged from larvae from Kerala, South India Virol J 2010 7 189 10.1186/1743-422X-7-189 20704755 PMC2928196

[B9] Tamura K Peterson D Peterson N Stecher G Nei M Kumar S MEGA5: Molecular Evolutionary Genetics Analysis using Maximum Likelihood, Evolutionary Distance, and Maximum Parsimony Methods Molecular Biology and Evolution 2011 28 2731 2739 10.1093/molbev/msr121 21546353 PMC3203626

[B10] Librado P Rozas J DnaSP v5: A software for comprehensive analysis of DNA polymorphism data Bioinformatics 2009 25 1451 1452 10.1093/bioinformatics/btp187 19346325

[B11] Powers AM Brault AC Tesh RB Weaver SC Re-emergence of chikungunya and O’nyong-nyong viruses: evidence for distinct geographical lineages and distant evolutionary relationships J Gen Virol 2000 81 471 479 10644846 10.1099/0022-1317-81-2-471

[B12] Tajima F Statistical methods to test for nucleotide mutation hypothesis by DNA polymorphism Genetics 1989 123 585 595 2513255 10.1093/genetics/123.3.585 PMC1203831

[B13] Fu YX Li WH Statistical tests of neutrality of mutations Genetics 1993 133 693 709 8454210 10.1093/genetics/133.3.693 PMC1205353

[B14] Huson DH Bryant D Application of Phylogenetic Networks in Evolutionary Studies Mol Biol Evol 2006 23 2 254 267 16221896 10.1093/molbev/msj030

[B15] Arankalle VA Shrivastava S Cherian S Gunjikar RS Walimbe AM Jadhav SM Sudeep AB Mishra AC Genetic divergence of Chikungunya viruses in India (1963–2006) with special reference to the 2005–2006 explosive epidemic J Gen Virol 2007 88 1967 1976 10.1099/vir.0.82714-0 17554030

[B16] Naresh Kumar CVM Anthony Johnson Am Sai Gopal DVR Molecular characterization of chikungunya virus from Andhra Pradesh, India & phylogenetic relationship with Central African isolates Ind J Med Res 2007 126 534 540 18219080

[B17] Tsetsarkin KA Chen R Leal G Forrester N Higgs S Huang J Weaver SC Chikungunya virus emergence is constrained in Asia by lineage-specific adaptive landscapes Proc Natl Acad Sci U S A 2011 108 7872 7877 10.1073/pnas.1018344108 21518887 PMC3093459

[B18] Grandadam M Caro V Plumet S Thiberge J-M Souarès Y Failloux A-B Tolou HJ Budelot M Cosserat D Leparc-Goffart I Desprès P Chikungunya virus, Southeastern France Emerg Infect Dis 2011 17 910 913 10.3201/eid1705.101873 21529410 PMC3321794

[B19] Sumathy K Ella KM Genetic diversity of Chikungunya virus, India 2006–2010: Evolutionary dynamics and serotype analyses J Med Virol 2012 84 462 470 10.1002/jmv.23187 22246833

[B20] Cui J Gao M Ren X Phylogeny and homologous recombination in Chikungunya viruses Infect Genet Evol 2011 11 1957 1963 10.1016/j.meegid.2011.08.026 21925290

[B21] Kumari R Kumar K Chauhan LS First dengue virus detection in Aedes albopictus from Delhi, India: Its breeding ecology and role in dengue transmission Trop Med Int Health 2011 16 949 954 10.1111/j.1365-3156.2011.02789.x 21668590

[B22] Tsetsarkin KA McGee CE Volk SM Vanlandingham DL Weaver SC Higgs S Epistatic roles of E2 glycoprotein mutations in adaption of Chikungunya virus to Aedes albopictus and Ae. aegypti mosquitoes PLoS One 2009 4 e6835 10.1371/journal.pone.0006835 19718263 PMC2729410

[B23] Ng LC Hapuarachchi HC Tracing the path of Chikungunya virus–evolution and adaptation Infect Genet Evol 2010 10 7 876 885 10.1016/j.meegid.2010.07.012 20654736

[B24] Hapuarachchi HC Bandara KB Sumanadasa SD Hapugoda MD Lai YL Lee KS Tan LK Lin RT Ng LF Bucht G Abeyewickreme W Ng LC Re-emergence of Chikungunya virus in South-east Asia: virological evidence from Sri Lanka and Singapore J Gen Virol 2010 91 Pt 4 1067 1076 19955565 10.1099/vir.0.015743-0

[B25] Cherian SS Walimbe AM Jadhav SM Gandhe SS Hundekar SL Mishra AC Arankalle VA Evolutionary rates and timescale comparison of Chikungunya viruses inferred from the whole genome/E1 gene with special reference to the 2005–07 outbreak in the Indian subcontinent Infect Genet Evol 2009 9 16 23 10.1016/j.meegid.2008.09.004 18940268

