# Supplementary material for: Genetic characterization of Chikungunya virus from New Delhi reveal emergence of a new molecular signature in Indian isolates
Source: Virol J. 2012 May 25;9:100. doi: 10.1186/1743-422X-9-100 (PMC3495852; doi:10.1186/1743-422X-9-100)
Supplement: Additional file 1 — Table S1. CHIKV sequences used in the study with accession number and collection date. Samples used in the study have been indicated by ‘♦’. [file 1743-422X-9-100-S1.doc]

| **Sample/State Name** | **Accession Number** | **Collection Date** |
| --- | --- | --- |
|  |  |  |
| IND-10-DEL1♦ | JF950631 | 2010 |
| IND-10-DEL2♦ | JF950632 | 2010 |
| IND-10-DEL10♦ | JF950617 | 2010 |
| IND-10-DEL100♦ | JF950643 | 2010 |
| IND-10-DEL102♦ | JF950642 | 2010 |
| IND-10-DEL103♦ | JF950621 | 2010 |
| IND-10-DEL104♦ | JF950622 | 2010 |
| IND-10-DEL106♦ | JF950637 | 2010 |
| IND-10-DEL108♦ | JF950623 | 2010 |
| IND-10-DEL11♦ | JF950634 | 2010 |
| IND-10-DEL110♦ | JF950639 | 2010 |
| IND-10-DEL113♦ | JF950638 | 2010 |
| IND-10-DEL119♦ | JF950624 | 2010 |
| IND-10-DEL12♦ | JF950635 | 2010 |
| IND-10-DEL123♦ | JF950641 | 2010 |
| IND-10-DEL14♦ | JF950636 | 2010 |
| IND-10-DEL15♦ | JF950647 | 2010 |
| IND-10-DEL17♦ | JF950646 | 2010 |
| IND-10-DEL19♦ | JF950645 | 2010 |
| IND-10-DEL20♦  IND-10-DEL3♦ | JF950618  JF950613 | 2010  2010 |
| IND-10-DEL4♦ | JF950648 | 2010 |
| IND-10-DEL5♦ | JF950614 | 2010 |
| IND-10-DEL52♦ | JF950644 | 2010 |
| IND-10-DEL6♦ | JF950615 | 2010 |
| IND-10-DEL75♦ | JF950619 | 2010 |
| IND-10-DEL8♦ | JF950616 | 2010 |
| IND-10-DEL81♦ | JF950640 | 2010 |
| IND-10-DEL9♦ | JF950633 | 2010 |
| IND-10-DEL91♦ | JF950620 | 2010 |
| IND-10-DEL42♦ | JF950625 | 2010 |
| IND-10-DEL48♦ | JF950626 | 2010 |
| IND-10-DEL84♦ | JF950627 | 2010 |
| IND-10-DEL88♦ | JF950628 | 2010 |
| IND-10-DEL107♦ | JF950629 | 2010 |
| IND-10-DEL109♦ | JF950630 | 2010 |
| Karnataka | EF027135.1 | 2006 |
| Karnataka | GQ996377.1 | 2008 |
| Karnataka | GQ996376.1 | 2008 |
| Karnataka | GQ996374.1 | 2008 |
| Karnataka | GQ996375.1 | 2008 |
| Karnataka | GQ996373.1 | 2008 |
| Karnataka | GQ996372.1 | 2008 |
| Karnataka | GQ996371.1 | 2008 |
| Karnataka | GQ996370.1 | 2008 |
| Karnataka | FJ617290.1 | 2008 |
| Karnataka | FJ617289.1 | 2008 |
| Nagpur | GQ996379.1 | 2006 |
| Nagpur | GQ996378.1 | 2006 |
| Maharashtra | AY424803.1 | 2003 |
| IND-00-MH4 | EF027139 | 2000 |
| IND-06-MH2 | EF027136.1 | 2006 |
| IND-MH51 | FJ000067.1 | 2006 |
| IND-73-MH5 | EF027141.1 | 1973 |
| Uttar Pradesh | EU727245.1 | 2007 |
| Uttar Pradesh | EU727246.1 | 2007 |
| Uttar Pradesh | EU727247.1 | 2007 |
| Uttar Pradesh | EU727248.1 | 2007 |
| Uttar Pradesh | EU727249.1 | 2007 |
| Kerala | GU562826.1 | 2009 |
| Kerala | GU562828.1 | 2009 |
| Kerala | GU562830.1 | 2009 |
| Kerala | GU562829.1 | 2009 |
| Kerala | GU562827.1 | 2009 |
| Kerala | EU372006.1 | 2007 |
| Kerala | FJ000066.1 | 2006 |
| Kerala | GQ428210.1 | 2006 |
| Kerala | GQ428211.1 | 2006 |
| Kerala | FJ617288.1 | 2008 |
| Kerala | FJ617287.1 | 2008 |
| Kerala | FJ617286.1 | 2008 |
| Kerala | FJ617285.1 | 2008 |
| Kerala | FJ617284.1 | 2008 |
| Kerala | FJ617283.1 | 2008 |
| Kerala | FJ617282.1 | 2008 |
| Kerala  Kerala | EU170527.1  EU170526.1 | 2007  2007 |
| Kerala | EU170525.1 | 2007 |
| Kerala | EU170524.1 | 2007 |
| Kerala | EU170523.1 | 2007 |
| Kerala | EF555197.1 | 2006 |
| Kerala | EF555196.1 | 2006 |
| Kerala | EU350536.1 | 2007 |
| Kerala | EU350535.1 | 2007 |
| Kerala | EU350534.1 | 2007 |
| Kerala | EU350533.1 | 2007 |
| Kerala | EU350532.1 | 2006 |
| Kerala | EU288003.1 | 2007 |
| Kerala | EU288002.1 | 2007 |
| Kerala | EU288001.1 | 2007 |
| Kerala | EU288000.1 | 2007 |
| Kerala | EU287999.1 | 2007 |
| Kerala | EU287998.1 | 2007 |
| Kerala | EU287997.1 | 2007 |
| Kerala | EU287996.1 | 2007 |
| Kerala | EU287995.1 | 2007 |
| Kerala | EU287994.1 | 2007 |
| IND-GJ53 | FJ000065.1 | 2006 |
| IND-GJ51 | FJ000064.1 | 2006 |
| IND-GJ52 | FJ000062.1 | 2006 |
| Gujarat | JF272479.1 | 2006 |
| Gujarat | JF274082.1 | 2006 |
| Gujarat | HM045794.1 | 2006 |
| Andhra Pradesh | GQ229489.1 | 2009 |
| Andhra Pradesh | GQ229488.1 | 2009 |
| Andhra Pradesh | GQ229487.1 | 2008 |
| Andhra Pradesh | GQ229486.1 | 2009 |
| Andhra Pradesh | FJ705371.1 | 2008 |
| Andhra Pradesh | FJ705370.1 | 2008 |
| Andhra Pradesh | FJ705369.1 | 2008 |
| Andhra Pradesh | FJ432665.1 | 2008 |
| Andhra Pradesh | EU886198.1 | 2006 |
| Andhra Pradesh | EF027134.1 | 2006 |
| Andhra Pradesh | HQ529779.1 | 2009 |
| Andhra Pradesh | HQ529777.1 | 2008 |
| Tamil Nadu | EF027138.1 | 2006 |
| Puducherry | EF555200.1 | 2006 |
| Puducherry | EF555199.1 | 2006 |
| Puducherry | EF555198.1 | 2006 |
| West Bengal | HM045813.1 | 1963 |
| Indonesia (Asia) | EU192143.1 | 2007 |
| RSU1 (Asia) | HM045797.1 | 1985 |
| Tanzania (ECSA) | HM045811.1 | 1953 |
| S27 (ECSA) | AF369024.2 | - |
| Senegal (Africa) | HM045817.1 | 2005 |
| Senegal (Africa) | HM045816.1 | 1966 |
| O'Nyong-nyong | M20303.1 | - |
